# Towards a Broad-Persistent Advising Approach for Deep Interactive Reinforcement Learning in Robotic Environments

**DOI:** 10.3390/s23052681

**Published:** 2023-03-01

**Authors:** Hung Son Nguyen, Francisco Cruz, Richard Dazeley

**Affiliations:** 1 School of Information Technology, Deakin University, Geelong 3220, Australia; 2 School of Computer Science and Engineering, University of New South Wales, Sydney 2052, Australia; 3 Escuela de Ingeniería, Universidad Central de Chile, Santiago 8330601, Chile

**Keywords:** reinforcement learning, deep reinforcement learning, interactive reinforcement learning, persistent advice, broad-persistent advising

## Abstract

Deep Reinforcement Learning (DeepRL) methods have been widely used in robotics to learn about the environment and acquire behaviours autonomously. Deep Interactive Reinforcement 2 Learning (DeepIRL) includes interactive feedback from an external trainer or expert giving advice to help learners choose actions to speed up the learning process. However, current research has been limited to interactions that offer actionable advice to only the current state of the agent. Additionally, the information is discarded by the agent after a single use, which causes a duplicate process at the same state for a revisit. In this paper, we present Broad-Persistent Advising (BPA), an approach that retains and reuses the processed information. It not only helps trainers give more general advice relevant to similar states instead of only the current state, but also allows the agent to speed up the learning process. We tested the proposed approach in two continuous robotic scenarios, namely a cart pole balancing task and a simulated robot navigation task. The results demonstrated that the agent’s learning speed increased, as evidenced by the rising reward points of up to 37%, while maintaining the number of interactions required for the trainer, in comparison to the DeepIRL approach.

## 1. Introduction

Robot development has achieved big steps toward improvement and has gained more attention in recent years. This success has not only come from industrial areas, where robots are gradually replacing humans [1], but also in the domestic areas. Their presence in domestic environments is still limited, mainly due to the presence of many dynamic variables [2] and safety requirements [3]. Intelligent robots in the future should be able to know and detect users, learn action objects, select opportunities, and learn to behave in domestic scenarios. To successfully perform these complex tasks, robots face many challenges such as pattern recognition, navigation, and object manipulation, all in different environmental conditions. That is, robots in the domestic environment need to be able to continuously acquire and learn new skills.

Reinforcement Learning (RL) is a method used for a robot controller in order to learn the optimal policy through interaction with the environment, through trial and error [4]. The use of RL in previous works showed that there is great potential for using RL in robots [2,5,6]. Especially, Deep Reinforcement Learning (DeepRL) has also achieved promising results in manipulation skills [7,8], how to grasp, as well as legged locomotion [9]. However, there is an open issue relating to the performance in the RL and DeepRL algorithms, which is the excessive time and resources required by the agent to achieve acceptable outcomes [10,11]. The larger and more complex the state space is, the more computational costs will be spent to find the optimal policy.

Among the different approaches to speed up this process, there is one promising method named Interactive Reinforcement Learning (IRL), which can improve the convergence speed and has shown its feasibility [12]. IRL allows a trainer to give advice or evaluate a learning agent’s behaviour [13], help the agent shape the exploration policy, and reduce the search space in the early stages. Combining IRL with DeepRL results in the model of Deep Interactive Reinforcement Learning (DeepIRL), which can be used in continuous space with improved learning speed [14]. However, current techniques using DeepIRL allow trainers to evaluate or recommend actions based only on the current state of the environment. The advice from the trainer is discarded after a single use, which leads to a duplicate process at the same state for a revisit.

This work introduces the Broad-Persistent Advising (BPA) approach for DeepIRL to provide the agent a method for information retention and reuse of previous advice from a trainer. The information will be efficiently retained, thereby facilitating expedited progress in the machine learning process. This approach includes two components: generalisation and persistence. This paper employed the *k*-means algorithm as a generalisation method to represent continuous space in storable data and implemented a Probabilistic Policy Reuse (PPR) sustainability model based on prior research [12]. To assess the effectiveness of the proposed approach, evaluations were conducted in both a conventional environment and a simulated robot one. Moreover, various advisor models were surveyed and compared for a comprehensive evaluation. In short, agents using the BPA approach have better results than their non-using counterparts while keeping the number of interactions required for the trainer.

The remaining sections of this paper are organised as follows: Section 2 presents a review of related literature, while Section 3 presents the proposed scheme. The experiments and results are discussed in Section 4 and Section 5, and finally, Section 6 presents the conclusion of the study.

## 2. Preliminary

### 2.1. Deep Reinforcement Learning

Reinforcement Learning (RL) is a branch of machine learning in which artificially intelligent agents learn behaviours by interacting with their surroundings [4]. Reinforcement learning tools learn through trial and error by repeatedly interacting with the surrounding environment and learning which actions will and which actions will not produce the expected results.

RL is appropriate for studying tasks that may be modelled as Markov Decision Processes (MDPs) [4,15,16]. An MDP is specified by the tuple (*S*, *A*, *T*, *R*, γ), where *S* is a finite set of states in the environment, *A* is a set of actions available in each state, *T* is the transition function *T*: $S_{n}\times A\to S_{n+1}$, *R* is the reward function *R*: S×A→R, and γ is a discount factor, which is 0≤γ≤1.

In the RL setup, a machine-learning-algorithm-controlled agent observes a state $s_{t}$ from its environment at time step *t*. In state $s_{t}$, the agent communicates with the environment by performing action $a_{t}$. Then, the agent moves to a new state $s_{t+1}$ and receives reward $r_{t+1}$ as feedback from the environment based on the previous state and the chosen action. Therefore, the reward collected by policy π at time step *t* is shown in Equation (1):

(1) $$r_{t}+\gamma r_{t+1}+\gamma^{2}r_{t+2}+\ldots =\sum_{k=0}\gamma^{k}r_{t+k}$$

where $r_{t}$ is the reward at time step *t*. The discount rate γ stands for the importance of rewards in the future. The agent’s aim is to find a policy π that maximises the anticipated profit (reward).

In conventional RL algorithms, most of the time, only MDP with discrete state and action spaces is considered. However, in many real-world applications, the state space is not really discrete, but rather a continuous domain [17]. Therefore, to be usable in the continuous state space, neural networks are also considered as function approximators, which are especially useful in RL when the state space or action space is too broad to fully comprehend [18,19,20]. Neural nets can discover ways to map states to values in this way. When the problem state space is too big or considered as a continuous space, we cannot use a lookup table to store and update all possible states and actions. In that case, one alternative is to train a neural network with samples from the state and the environment and expect it to predict the value of the next action as the target in RL. More formally, we used a neural network to approximate the optimal action–value function, which is the maximum sum of rewards in Equation (2):

(2) $$Q^{*}\left(s_{t},a_{t}\right)=\max_{\pi }E\left[\sum_{0}\gamma^{k}r_{t+k}|s_{t}=s,a_{t}=a,\pi \right])$$

A vast variety of recent advanced robot applications have been accomplished using deep reinforcement learning to teach agents complex activities including cube play [21], ambidextrous robot gripping [7], categorised objects [14], and the table-cleaning task [22]. For instance, Cruz et al. [22] used an associative neural architecture to learn the available action possibilities of the agents with the objects in the current context. Levine et al. [23] proposed a learning-based approach to hand–eye coordination for robotic grasping from monocular images using a large Convolutional Neural Network (CNN) to learn the way to grasp objects.

### 2.2. Reinforcement Learning with Interactive Feedback

**Figure 1:**
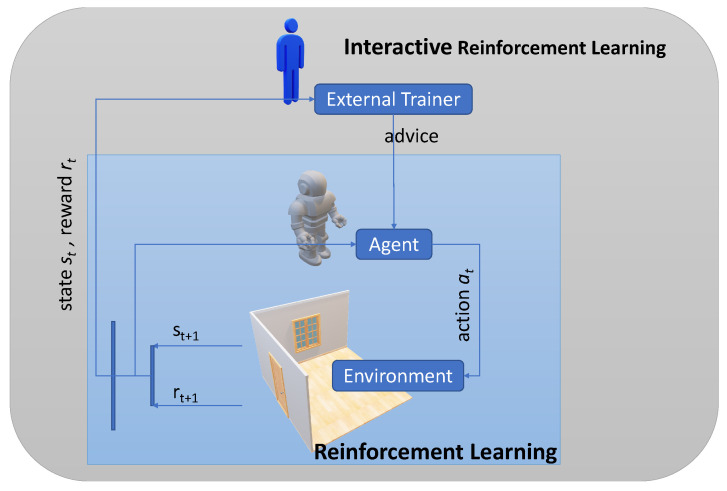
At state $s_{t}$, the agent performs action $a_{t}$, obtains reward $r_{t+1}$, and reaches the next state $s_{t+1}$ in conventional RL. In the IRL model, the involvement of an external trainer gives the agent more options to chose which action to perform next in the following iteration.

In Interactive Reinforcement Learning (IRL), there is an external trainer involved in the agent’s learning process [13,20,24,25]. Figure 1 depicts the IRL solution, which includes an advisor, who observes the learning process and offers guidance on the way to improve the decision-making [26]. The advisor can be an expert human or an artificial agent.

Adaptive agent behaviour is needed in domestic environments. IRL enables a parent-like tutor to facilitate learning by providing useful guidance in some particular situation, allowing the apprenticeship process to be accelerated. In contrast to an agent exploring completely autonomously, this makes for a smaller search space and, hence, quicker learning of the mission [27].

When operating alone, the next step is chosen by selecting the better-known action at the current time, defined by the highest state–action pair, while IRL accelerates the learning process by incorporating additional guidance into the apprenticeship loop. Using IRL, a trainer with prior experience of the target goal is required [28].

There is a difference between the two main methods dedicated to feedback learning: reward shaping and policy shaping. While in reward shaping, external trainers can assess the quality of the actions performed by the RL agent, as good or bad [28,29], using policy shaping, the actions proposed by the RL agent can be replaced by more appropriate actions selected by the external trainer before implementation [30,31].

An open problem that can significantly affect the agent’s performance is inaccurate advice from the trainer [13], since a lack of accuracy and repetitive mistakes will result in a longer training time. Human advice, on the other hand, is not 100% correct [32]. When an advisor gives too much guidance, the agent will have limited experience in exploration because the trainer makes almost all of the decisions [33]. To address the problem, a prior study [34] applied to the agent a strategy of discarding or refusing advice after an amount of time, endowing the agent with the ability to work with potentially incorrect information.

## 3. A Broad-Persistent Advising Approach

In this section, we give more details about the proposed Broad-Persistent Advising (BPA) approach, which includes a generalisation model along with a persistent approach. These details are described next.

### 3.1. Persistent Advice

A recent study [34] suggested a permanent agent that records each interaction and the circumstances around particular states. The actions are re-picked when the conditions are met again in the future. As a consequence, the recommendations from the advisor are used more effectively, and the agent’s performance improves. Furthermore, as the training step has no need to provide advice for each repeated state, less interaction with the advisor is required. However, in this experiment, we limited the research to keeping the same number of interactions with the trainer to investigate the effectiveness of the BPA approach in the continuous domain.

As previously mentioned, there is an issue relating to inaccurate advice. After a certain amount of time, a mechanism for discarding or ignoring advice is needed. Probabilistic Policy Reuse (PPR) is a strategy for improving RL agents that use advice [35]. Where various exploration policies are available, PPR uses probabilistic bias to decide which one to choose, with the intention of balancing between random exploration, the use of a guideline policy, and the use of the existing policy.

**Figure 2:**
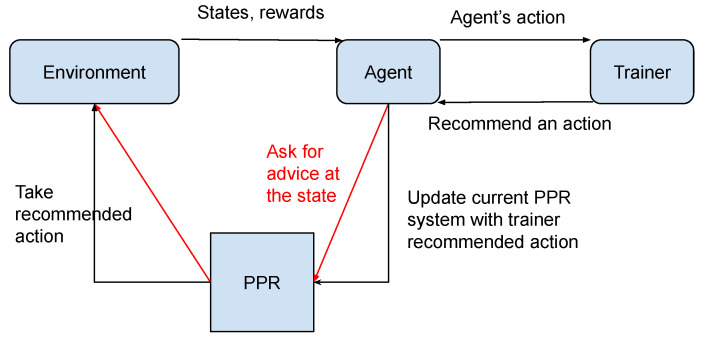
Process flow of an interactive reinforcement learning agent using the PPR system. After receiving advice from the trainer, the agent will store the action in the PPR model. In the iteration not receiving advice from the trainer, the agent will check and reuse the old advice (red arrows) from the past.

Figure 2 denotes an example of IRL using PPR. The advising user has the opportunity to engage with the agent at each time point. When there is an interaction, the model is updated. At the time advice is firstly recommended, it is assumed that the agent will carry out the suggested action, regardless of the setting of PPR. PPR is used at the time step when the agent did not receive advice from the trainer, the flow of which is denoted by red arrows. First, the agent’s policy is examined to see whether any advice is applicable to the existing state. If the current policy suggests an action, the action is taken as determined by the PPR selection policy.

### 3.2. Broad Advice

To use PPR, we need a system to store the used state–action pairs. When the agent arrives at a certain state at a time step, agents using PPR need to check with the system if this state has been suggested by the trainer in the past. If there is advice in the memory of the model, the agent can use the option to reuse the action. However, there is a problem when using PPR in infinite domains. We cannot build a system that stores state–action pairs with infinite state values. In addition, when the amount of states becomes too large in the space, which is equivalent to infinity, the possibility that agents revisit exactly the same state will be very small. Therefore, building this model will become cumbersome and inefficient in large spaces.

**Figure 3:**
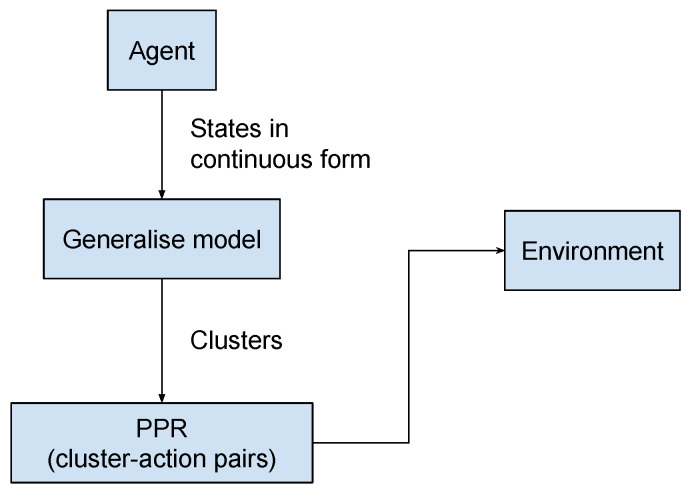
Broad advice transforms continuous states into finite clusters. Hence, the state–action pair becomes the cluster–action used in the PPR model.

BPA includes a model for clustering states and then building a system for cluster–action pairs instead of traditional state and action pairs. The proposed model is shown in Figure 3. When the agent receives current state information from the environment and it does not receive any advice from the trainer, the agent will use PPR by injecting the state into the generalisation model and defining its cluster. Then, it proceeds to consider whether any advice pertains to the current cluster. If there was an action recommended in the past, the agent can reuse it with the PPR selection probability or use the default action as ϵ-greedy.

The generalisation model we used in this paper is the *k*-means algorithm. *k*-means is one of the most-popular clustering methods [36]. *k*-means is simple to implement, and its complexity scales well with a higher number of data. However, the user must decide on the number of clusters beforehand [37]. We used the elbow technique to specify the number of clusters [38]. It is the visual graphic approach that was generated from the Sum of Squares Error (SSE) computation. This technique is based on the idea that the number of clusters should be chosen so that adding another cluster does not cause significantly improved modelling. The early clusters will provide much information, but at some point, the marginal gain will drop drastically, giving the graph an angle. At this angle, the correct *k*-number of clusters is determined, thus called the “elbow criteria”. In other words, the value of k is chosen at the point where increasing k does not significantly decrease the value of the Sum of Squares Error (SSE).

## 4. Experimental Environments

### 4.1. Cart Pole Gym Environment

The deep reinforcement learning environment was implemented using the well-known library of AI gym environments [39]. First, we built the cart pole environment [40]. In this environment, there is a pole that is attached to a cart. The carriage can move by applying force to the left or right. The purpose of this problem is to prolong the time while avoiding the pole falling down. The terminal condition is that the pole deviates more than 15 degrees from the vertical or the wagon moves 2.4 units from the centre. The cart pole MDP is defined as follows:

- State: The state vector has a continuous representation with four attributes, which represent the cart position, cart velocity, pole angle, and pole velocity.
- Action: The cart can perform two actions on the track: go to the left or the right.
- Reward function: As long as the agent holds the pole in a vertical position, a reward equal to 1 is awarded, and if it drops or goes beyond the boundaries of the track, the reward is equal to 0.

**Figure 4:**
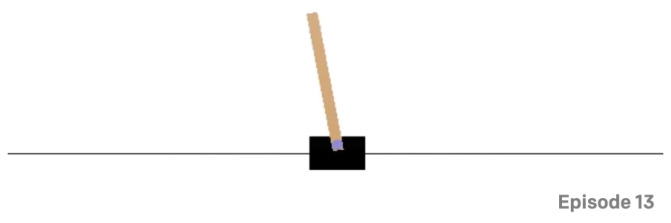
A graphical representation of the cart pole environment. The goal is to keep the pole balanced while applying forces to the carriage. The terminal condition is that the pole deviates more than 15 degrees from the vertical or the wagon moves 2.4 units from the centre.

Figure 4 below denotes a graphic of the cart pole in the AI gym environment.

### 4.2. Domestic Robot Environment

**Figure 5:**
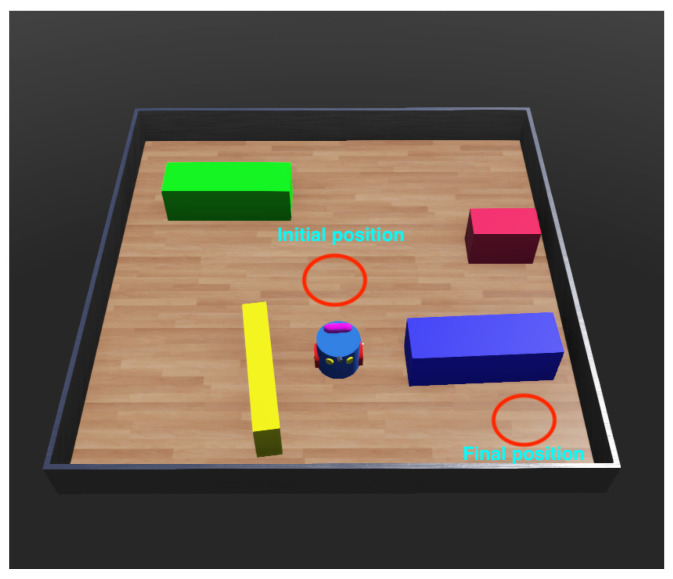
An example of the Webots environment with the initial position and final position. The robot has the goals of going from the initial position to the final position while avoiding obstacles. The robot will be returned to its initial position after any collision.

Additionally, we also built an environment for domestic robots using Webots. In this environment, the goal is to train the robot to go from the initial position to the target position. Figure 5 gives a graphic of our experimental environment in Webots.

The robot is equipped with distance sensors on its left and right eyes. The robot is completely unaware of its current position in the environment. The robot can only choose one of three actions: go straight at 3 m/s, turn left, or turn right. At each step, the robot will be deducted 0.1 points if it uses the action of turning left or right, while no points will be deducted if it chooses to go straight. This is to optimise the robot’s straight movement and avoid the robot running in circles by turning left or right continuously. The robot is equipped with a few touch sensors next to it, to detect the collision with the environment. The robot will be returned to its initial position and receive 100 penalty points every time it collides on the way. The robot does not know where the touch sensor is located relative to itself; the only information it receives is whether it is a collision with obstacles or not. When the robot goes to the finish position located in the lower-right corner of the environment, the robot is considered to have completed the task and will be rewarded 1000 points.

**Figure 6:**
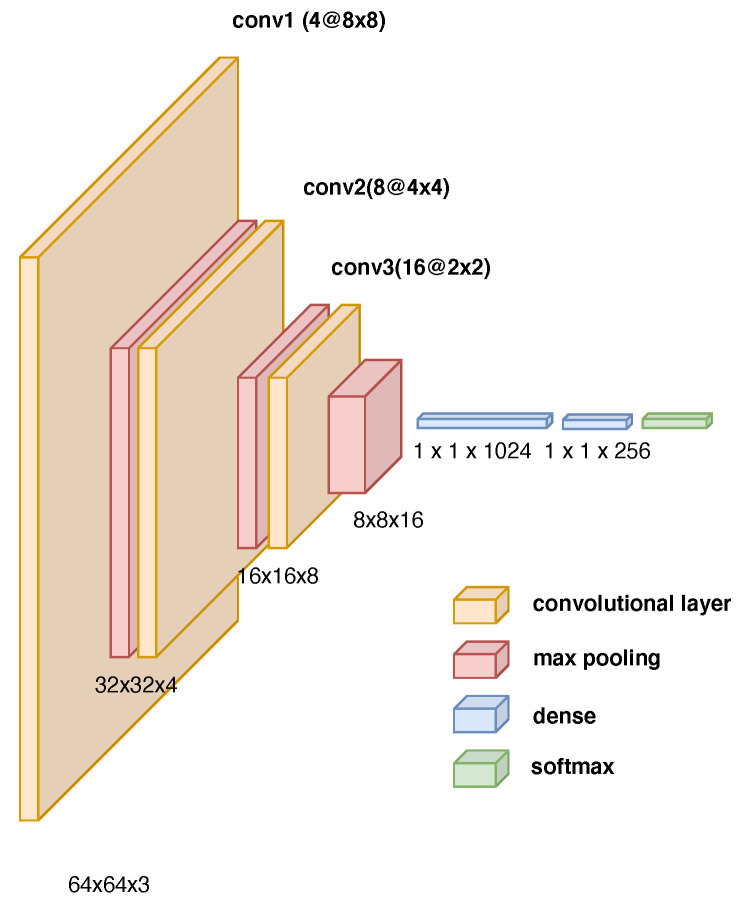
CNN architecture with a 64 × 64 RGB image as the input, three convolutional layers, three max-pooling layers, two dense fully connected layers, and a softmax function at the output.

To decide on the next action the robot will take, the robot’s supervisor will use the image taken from the top of the environment to enter the Convolutional Neural Network (CNN) system to decide. The CNN system built in this environment is a system whose input is 64 × 64 image RGB channels. This architecture was inspired by similar networks used in other DeepRL works [14,41,42,43,44]. In more detail, we used 4 kernels with a size of 8 × 8. The second layer has 8 kernels with a size of 4 × 4, and the last layer convolution is 16 kernels with a size of 2 × 2. Following each convolutional network layer is a 2 × 2 max-pooling layer. Finally, there is a flatten and dense layer with 256 neurons fully connected with the output layer. The network architecture is described in Figure 6.

The environment MDP is defined as follows:

- State: RGB image size 64 × 64 taken from the top of the environment.
- Action: Three actions: go straight at 3 mm/s, turn left, or turn right.
- Reward function: Turn left, right: −0.1; go straight: 0; collision: −100; reach final position: 1000.

### 4.3. Interactive Feedback

While the interactive agent’s human-related approach to learning is one of its greatest strengths, it may also be its greatest weakness [45,46,47]. Advice with good accuracy given in the proper time will help the agent greatly to increase the speed of finding the optimal solution. However, in the case when the agent only gives advice with low accuracy and at a high frequency, that not only does not help the agent, but also brings it to a dead end and is much more time-consuming than the non-interaction situation. Furthermore, human experiments are costly, time-consuming, have problems of repeatability, and can be difficult to recruit volunteers for. Therefore, during the early stages of developing the agent, we suggested that simulating human interactions would be much more convenient.

To compare the agent’s performance, information about the agent’s steps, rewards, and interactions were recorded. To identify the efficiency of BPA, we needed to test the experiment with three cases: no interactive action, interactive actions without BPA, and interactive actions with BPA.

Each use case of the simulated user will have different advice accuracy and frequency. Accuracy is a measure of the precision of advice provided by an advisor. When the advisor’s precision is high, the action will be proposed precisely as the advisor’s knowledge of the environment. On the contrary, the advisor will propose a non-optimal action based on what it knows about the environment. Frequency is the availability of the interaction of the advisor at the given time step. The higher the frequency is, the higher the advisor’s rate of giving advice to the agent is. The accuracy and frequency of three kinds of agents were used with the values described in Table 1. Optimistic simulated agents have 100% accurate advice and always provide advice at every time step. Realistic simulated agents use accuracy and frequency values from the results of a human trial [32,34]. The pessimistic value of the frequency is 0%; however, it works the same as in the case without interactive feedback. Therefore, we used half of the realistic values for the case with the least interaction of the advisor. The accuracy and frequency values of the advice were beyond the scope of this study.

The corresponding frequency and accuracy values above are pessimistic values, realistic values, and optimistic values, respectively.

Experiments were performed for each case, and the indicator of the amount of accumulated reward to achieve the optimal policy was recorded to compare the results between many approaches. The more reward the agent has, the better the result of the method is.

### 4.4. Generalised Model and Probabilistic Policy Reuse

**Figure 7:**
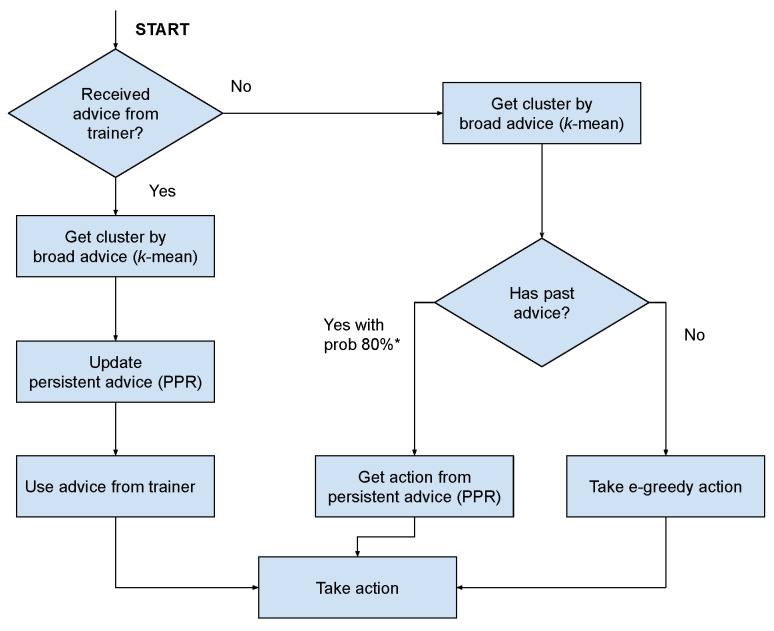
Flow of using broad-persistent advising. The agent will reuse previously obtained advice with an 80% chance (which decays over time) and perform its exploration policy for the remaining change (20%).

Next, we demonstrate the use of broad advice and persistent advice using Probabilistic Policy Reuse (PPR). The flow of using PPR is depicted in Figure 7. Initially, the agent reuses the action using PPR with a certain chance if the current state has been recommended by the trainer in the past. In this work, we used a chance value of 80%, which has also been used in previous research [34]. This probability decreased by 5% for each step. With the remaining 20%, the greedy action policy was selected.

Algorithm 1 shows the process flow for selecting an action using the BPA approach to assist a learning agent.
**Algorithm 1** Interactive reinforcement learning with BPA.   1:  Build *k*-means model with states from trainer   2:  Initialise environment selecting $s_{t}$   3:  **for all** (episodes) **do**
   4:        **repeat**
   5:              **if** (have feedback) **then**   6:                    Get recommended action $a_{t}$   7:                    Get cluster $c_{t}$ by using *k*-means   8:                    Add pair ($c_{t}$, $a_{t}$) to PPR   9:              **else**
 10:                    **if** (rand(0,1)<ϵ) **then**
 11:                           Get $c_{t}$ by using *k*-means
 12:                           **if** ($c_{t}$ is available in PPR) **then**
 13:                                 Get $a_{t}$ is reuse action from PPR
 14:                           **else**
 15:                                 Random action $c_{t}$ from environment
 16:                           **end if**
 17:                    **else**
 18:                           Choose action $a_{t}$ using π
 19:                    **end if**
 20:              **end if**
 21:              Perform action $a_{t}$
 22:              Observe next state $s_{t+1}$
 23:        **until** (*s* is terminal)
 24:        Update policy π
 25:  **end for**


The model was tested with the following agents listed below:

- Baseline reinforcement learning: The model is trained in a basic manner and collects information from the environment without using any interactive feedback or evaluation from the trainer. It was used as a benchmark.
- Non-persistent reinforcement learning: The agent is assisted by multiple types of users as mentioned before in Table 1. After taking the recommendation from the trainer and executing the action, the agent will discard the advice. When the agent comes to a similar state again in the future, it cannot recall the previous recommendation and performs an ϵ-greedy action instead.
- Persistent reinforcement learning: This agent is supported by a trainer and a PPR system. The trainer can suggest an action in each time step for the agent to take. If recommended, the learning agent will perform on that time step and retain the recommendation for reusing when it visits a similar state in the future. When an agent accesses a similar state it has previously suggested, it will perform that action with the probability determined by the PPR action selection rate.

## 5. Results

### 5.1. Cart Pole Domain

In this section, we proceed by displaying the results of the three types of agents proposed above: baseline RL for benchmarking, non-persistent RL, and persistent RL. For agents of the types non-persistent RL and persistent RL, we conducted tests at different frequencies and accuracies of the feedback, called the optimistic user, the realistic user, and the pessimistic user. The method for all the agents were tested with the same hyperparameters as follows: initial value of ϵ = 1, ϵ decay rate of 0.99, learning rate α = 0.01, and discount factor γ = 0.99 during 500 episodes. To better display the results, we computed the average value of the last 100 rewards instead of the current episode’s reward. We were inspired by the idea from an article with the same result for the cart pole environment [48].

**Figure 8:**
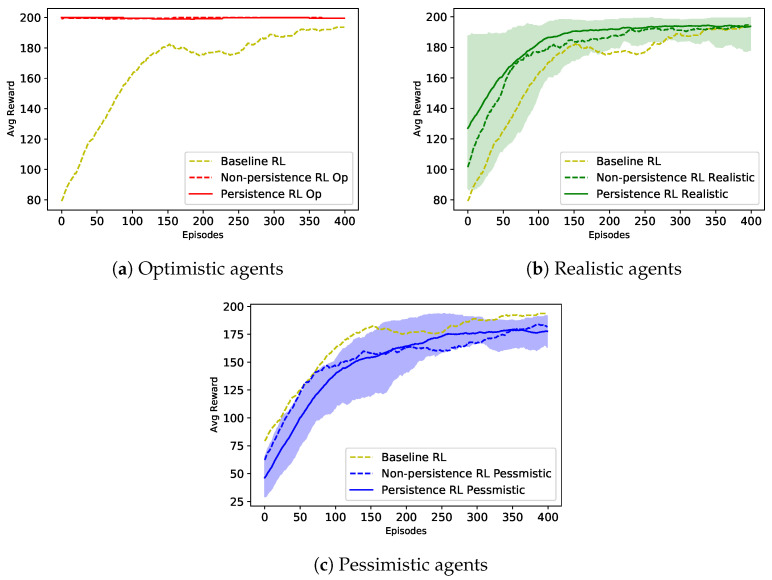
The comparison of the persistent agents, non-persistent agents, and baseline for each kind of agent in deep reinforcement learning built with the cart pole environment. The shaded area indicates the deviation between the minimum and maximum values of the agent value after multiple training times.

The results obtained are shown in Figure 8. The optimistic, realistic, and pessimistic agents were run five times and are represented by red, green, and blue lines, respectively. The shaded area indicates the standard deviation of the agent’s reward after multiple training times. Overall, all interactive agents outperformed the autonomous one (baseline RL in yellow), except the pessimistic agents. The agents that received advice from the instructor made fewer mistakes, especially in the early stages of the learning process, and could learn the task in fewer episodes. However, in this work, we wanted to compare pairs of non-persistent and persistent agents with the same style delivered advice to verify if the BPA approach implementation was indeed effective.

The agents assisted by optimistic advisors achieved the maximum score of the DeepIRL algorithms very early after a few episodes, given that the trainer always made decisions for the agent (100%), and this decision was absolutely correct (100%). In this experiment, the agents did not even have a chance to make their own decisions or use PPR; the trainer made all the decisions.

On the contrary, the agents supported by pessimistic users had different results, but in fact, neither of them could solve the problem. On many runs in both the non-persistent and persistent cases, the agent failed to achieve convergence. Both cases were considered worse than the baseline. This can be explained by the accuracy of the advice for the pessimistic agents being only 23.658%.

On the graph of the agents being helped by realistic trainers, we can see that using PPR produced slightly better results than the non-using counterpart. The persistent agents not only had a better initial reward, but also could achieve convergent results 100 episodes earlier than the non-persistent agents. The average reward points earned by a realistic agent with PPR were higher than 2.8%, as evidenced by an average of 183.65 points compared to an average of 178.9 points. This difference in the learning rates was due to the fact that the agent retained and reused the advice. In this experiment, the realistic agent had a 47.3% chance to interact with the trainer and the agent would withhold or not withhold the advice given by the trainer depending on whether it is a persistent agent or a non-persistent agent. However, the persistent agent retained and reused the advice with an 80% probability (decreasing over time) for any state in which it had received the advice in the past. As long as the stored advice was accurate enough, the persistent agent would learn faster because they used the advice more often.

In this experiment, we focused on implementing persistent advice in a continuous environment. The ratio of the number of interactions with our trainers remained the same: for example, 47.316% with the realistic agent. When receiving advice from the trainer, the agent will always prioritise executing this recommended action. Therefore, the number of interactions using the BPA method was equivalent to not using it. Table 2 shows the average number and percentage of interactions that occurred for each agent. Both the non-persistent and persistent agents used the interaction rate according to Table 1. We can see that the numbers of interactions of every pair of optimistic, realistic, and pessimistic agents were similar in the experiment.

**Figure 9:**
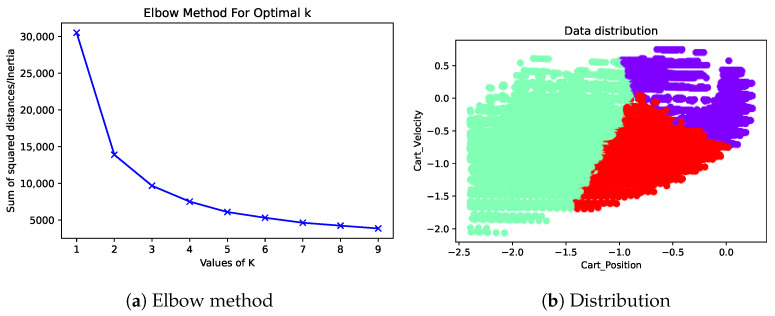
Total squared distance for the value of *k* from 1–9 and the distribution for 50,000 states with a value of *k* = 3 for the cart position and cart velocity attributes.

Figure 9a shows a graph using the elbow method to specify the number of clusters as a parameter of *k*-means, using the data of 50,000 states, the same number as the experiment for the cart pole, in the actual running environment. The elbow method showed the best value for using *k* at a value of three. Figure 9b displays the data distribution in the cart position and cart velocity attributes’ axes at a value *k* = 3.

### 5.2. Webots Domain

In this scenario, we focused only on examining the results for the realistic agent, because this can be transferred to the real-world scenarios in a more rational manner. The method was tested with the following hyperparameters: initial value of ϵ = 1, ϵ decay rate of 0.99, learning rate α = 0.01, and discount factor γ = 0.99 during 500 episodes. We used the average value of the last 100 rewards instead of the current reward only.

**Figure 10:**
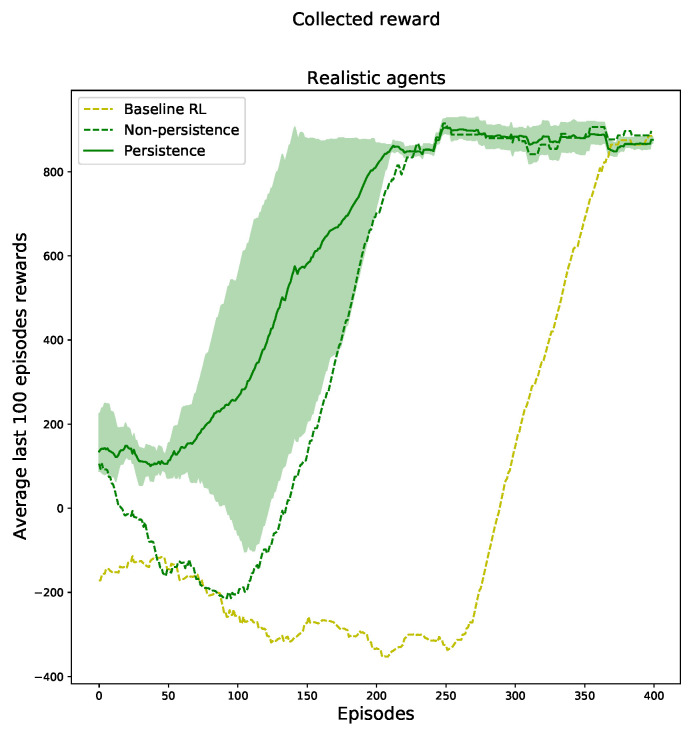
Result for deep reinforcement learning with the autonomous agent, the non-persistent agent, and the persistent agent built with the Webots domestic robot environment.

The results obtained are shown in Figure 10. The non-persistent RL agent is shown by a green dashed line, while the persistent RL agent is shown by a green solid line. The baseline RL is drawn with a yellow line used for benchmarking. Similar to the cart pole environment, both agents supported by the trainer, regardless of whether or not they used PPR, obtained better results than for the baseline RL. Then, the persistent agent achieved convergent results slightly earlier than its non-persistent counterpart. The average reward points obtained using PPR was 616, while the average reward points obtained without using PPR was 451, representing a significant increase of 37%. The trainer’s accuracy and frequency feedback was used the same as in the cart pole environment, so the results reflect the use in the domestic robot environment as well and not just an ideal hypothetical environment, such as the cart pole in the AI gym.

Table 3 shows the average number and percentage of interactions that occurred for each agent. We can see that the number of interactions was similar in the experiment.

**Figure 11:**
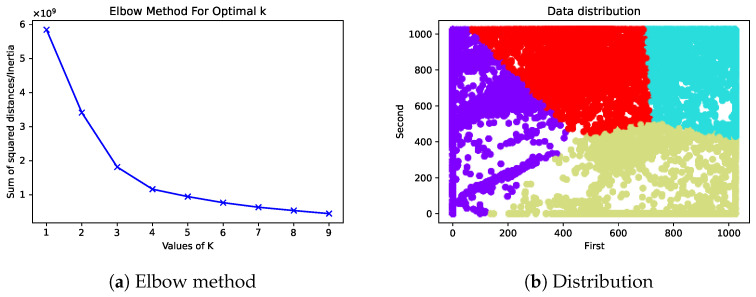
Total squared distance for a value of *k* from 1–9 and the distribution for 50,000 states with a value of *k* = 4 for two value attributes of the distance sensor.

Figure 11a shows a graph using the elbow method to specify the number of clusters as a parameter of *k*-means, using the data of 50,000 states in the actual running environment. The elbow method showed the best value for using *k* at a value of four. Figure 11b displays the data distribution in two axes of the distance sensor value at the value *k* = 4.

## 6. Conclusions and Future Work

In this work, we proposed BPA, a broad-persistent advising approach to implement the use of PPR and generalised advice in continuous state environments. Moreover, we also performed a comparison between autonomous DeepRL, DeepIRL without the BPA approach, and DeepIRL with the BPA approach. Two environments were tested to investigate the impact the BPA approach had on the performance.

Overall, the results obtained showed that the BPA approach with *k*-means as a generalised model and PPR as a model of persistence performed slightly faster and obtained convergence earlier up to 100 episodes when the advice was withheld. There was a 37% increase in the reward points observed in the robot simulator environment. Moreover, higher accuracy for the advice and a longer time to retain it significantly increased the learning speed. Our research demonstrated that implementing PPR in a continuous state space environment is feasible and effective. Furthermore, *k*-means as broad advice inherited advantages due to its characteristics, such as the fast running time and scalability over a large state space, making it suitable for real-world environments.

In addition to *k*-means, exploring alternative generalisation models is necessary to gain a more comprehensive understanding of their effectiveness when using PPR in a reinforcement learning environment. Conducting thorough research is essential to determine the most-suitable generalisation approach. The accuracy of the generalisation model has a considerable impact on the speed and convergence of the IRL model, because agents hardly achieve convergent results when faced with numerous incorrect suggested actions. Additionally, we suggest reducing the number of interactions with the trainer by reusing actions in the persistent model more frequently. If the agent reaches a new state already in memory, it immediately reuses the recommended action without consulting the trainer. However, this approach should only be applied when the generalisation model is good enough. Furthermore, we plan to transfer and evaluate our proposed approach in a real-world setting that involves human and robot interaction.

## Figures and Tables

**Table 1:** The three simulated users designed for the experiments. These users are not intended to be compared against each other, rather with a persistent counterpart.

Agent	Frequency	Accuracy
Pessimistic Advisor	23.658%	47.435%
Realistic Advisor	47.316%	94.87%
Optimistic Advisor	100%	100%

**Table 2:** The average number of interactions in the experiment for each kind of agent and the percent compared with the total steps taken.

Agent	Interaction	
	Non-Persistent	Persistent
Optimistic Advisor	99,796 (100%)	99,846 (100%)
Realistic Advisor	40,976 (47.15%)	41,832 (47.1%)
Pessimistic Advisor	18,034 (23.62%)	16,685 (23.76%)

**Table 3:** The average number of interactions in the experiment and the percent compared with the total steps taken for the realistic agent in the Webots environment.

Agent	Interaction	
	Non-Persistent	Persistent
Realistic Advisor	9077 (47.64%)	8241 (47.18%)

## Data Availability

Not applicable.
